# Multimodal Evaluation of Three NiTi Rotary Systems: Clinical Simulation, Mechanical Testing, and Finite Element Analysis

**DOI:** 10.3390/dj13080368

**Published:** 2025-08-15

**Authors:** Jesus A. Aparicio, Pedro M. Mendez S, Giulia Malvicini, Simone Grandini, Carlo Gaeta, Ana Paula García Guerrero, Kristel Lidice Miranda Robles, José Aranguren, Alejandro R. Pérez

**Affiliations:** 1 Graduate Endodontics Program, School of Dentistry, Universidad Catolica de Santiago de Guayaquil (UCSG), Guayaquil 090615, Ecuador; jesus.aparicio01@cu.ucsg.edu.ec (J.A.A.); yara_anna.garcia@cu.ucsg.edu.ec (A.P.G.G.); kristel.miranda@cu.ucsg.edu.ec (K.L.M.R.); 2 Surpreendente Research Group, 4400-239 Vila Nova de Gaia, Portugal; od.pedromendez@gmail.com; 3 Unit of Endodontics and Restorative Dentistry, Department of Medical Biotechnologies, 53100 Siena, Italy; giulia.malvicini@student.unisi.it (G.M.); grandini@unisi.it (S.G.); odontoiatriagaeta@libero.it (C.G.); 4 Department of Endodontics, Rey Juan Carlos University, 28922 Madrid, Spain; josearanguren@hotmail.com

**Keywords:** cyclic fatigue, finite element analysis, nickel–titanium rotary instruments, torsional resistance, 3D-printed root canal replicas

## Abstract

**Objectives:** This study aimed to compare the clinical durability, mechanical performance, and stress behavior of three NiTi rotary systems—BlueShaper (Blue), BlueShaper Pro (Dual Wire), and BlueShaper Gold (fully gold-treated NiTi)—through a multimodal evaluation that included simulated instrumentation in 3D-printed replicas, mechanical testing, and finite element analysis (FEA). **Methods:** Sixty instruments (n = 20 per group) were tested. Simulated canal preparation was conducted in standardized 3D-printed mandibular molars with a 40° mesial root curvature until fracture occurred. Mechanical tests included torsional and flexural loading using a universal testing machine and stainless steel blocks with a standardized 40° curvature. FEA simulations evaluated von Mises stress, shear stress, total deformation, cyclic fatigue behavior, and contact pressure between the instrument and canal wall. **Results:** BlueShaper Gold prepared an average of 7.5 canals before fracture, followed by BlueShaper Pro (5.67 canals) and Blue (5.00 canals) (*p* < 0.001). Gold exhibited the highest torsional resistance (6.08 ± 3.08 N) and the longest fatigue life (325 ± 55.7 cycles), with the lowest von Mises stress and damage factor in FEA. BlueShaper Pro showed the longest time to fracture in mechanical testing (73.85 ± 7.10 s) and balanced mechanical behavior. Blue demonstrated the lowest performance across most parameters, including the shortest fatigue life and highest stress concentration. **Conclusions:** BlueShaper Gold exhibited the highest mechanical strength and fatigue resistance. BlueShaper Pro demonstrated the longest fatigue life and balanced mechanical behavior. Blue showed the lowest performance across most parameters. The strong correlation among clinical, mechanical, and FEA data reinforces the critical role of alloy composition in determining instrument durability, even when design remains constant.

## 1. Introduction

Nickel–titanium (NiTi) rotary instruments have become the cornerstone of contemporary endodontic shaping techniques due to their superior flexibility, shape memory, and fatigue resistance compared to traditional stainless steel files [1]. Over the past two decades, the evolution of NiTi instruments has been driven not only by design and cross-sectional geometry refinements but also by advances in thermomechanical treatments that alter the alloy’s crystalline structure [2,3,4]. These processes, like M-Wire, Blue, and Gold adjust the balance between martensite and austenite, lower the elastic modulus and improve performance, and modify the phase transformation behavior and, consequently, the mechanical performance of the instruments under clinical loads [5,6]. The resulting surface colors reflect oxide layer differences and indicate thermal conditioning, not changes in alloy composition [7].

Numerous studies have explored the influence of design variables such as taper, tip diameter, and cross-sectional design on performance outcomes, including cyclic fatigue resistance, torsional strength, and shaping ability [4,8,9,10,11,12,13]. In parallel, an increasing number of investigations have focused on the effects of alloy treatment, showing that heat-treated files exhibit distinct stress responses and failure mechanisms [1,14,15]. However, many of these studies evaluate instruments with simultaneous differences in both geometry and metallurgy, making it difficult to attribute the observed differences solely to the alloy properties [1]. Thermomechanical treatments applied to NiTi instruments alter phase transformation temperatures and increase martensitic content at body temperature, which enhances flexibility and fatigue resistance without changing the base alloy composition [1,7].

Furthermore, although mechanical testing methods, such as torsional resistance and cyclic fatigue testing, are widely accepted for comparing NiTi files, they only partially explain how instruments perform under dynamic clinical conditions [16]. Finite element analysis (FEA) has emerged as a complementary tool to simulate internal stress distribution and predict failure zones, while 3D-printed replicas of root canals offer a standardized platform to simulate instrumentation in anatomically relevant models [16,17,18,19]. This process involves breaking down the intricate structure of a rotary file into numerous small, simple components, making it easier to compute each deformation (strain and stress) compared to analyzing the entire large structure at once. Nevertheless, this approach demands extensive data input and results in longer processing times [20].

The use of 3D-printed teeth in endodontic research has gained significant traction due to the ability to standardize canal anatomy and eliminate the variability inherent in natural teeth [21,22,23]. These models, typically reconstructed from micro-CT scans, allow reproducible testing conditions for evaluating shaping ability, fatigue resistance, and instrumentation efficacy [24]. Studies have demonstrated that 3D-printed canals provide reliable simulation of curved and complex anatomies and are suitable for comparing file performance under controlled conditions [22,24].

Despite these methodological advances, there is still a lack of integrative studies that combine all three approaches—mechanical testing, clinical simulation, and computational modeling—to comprehensively evaluate how different alloys influence instrument behavior in clinically relevant situations [25].

This knowledge gap is particularly relevant for instruments with identical geometries made from different thermally treated NiTi alloys. Understanding how metallurgy alone affects clinical performance and could lead to more informed instrument selection based on canal anatomy, case difficulty, and the risk of instrument separation.

Therefore, this study aimed to evaluate and compare the mechanical performance, fatigue resistance, and the dentin–instrument stress interaction of three geometrically identical NiTi rotary instruments manufactured with different thermal treatments: a conventional Blue-wire alloy, a full Gold-wire alloy, and a hybrid Dual Wire configuration. The evaluation used a multimodal methodology, including mechanical testing under torsional and flexural loads, simulated instrumentation in 3D-printed molars with standardized curvature, and finite element analysis to assess internal stress, deformation, and contact forces transmitted to dentinal walls. The null hypothesis of this study was that thermal treatment would not significantly affect the mechanical performance, fatigue resistance, or stress distribution of NiTi rotary instruments when the instrument geometry is held constant.

## 2. Materials and Methods

### 2.1. Sample Size Calculation

The present in vitro study was approved by the local Ethics Committee of the University Hospital of Siena, (Siena, Italy), Area Vasta Toscana Sud Est (protocol number: 7/2021; approval date: 17 November 2021).

The required sample size was determined using G*Power software (version 3.1), applying a two-tailed independent *t*-test with an expected effect size of 1.2, a significance level of 0.05, and a statistical power of 0.80. This calculation yielded a minimum of 12 samples per group. To enhance statistical robustness and account for potential outliers, 20 instruments were included per system, for both the physical fracture tests and clinical testing using 3D-printed replicas.

### 2.2. Tooth Model Preparation

A mandibular molar with a Vertucci Type IV configuration in the mesial root was selected and scanned using high-resolution micro-computed tomography (Micro-CT) (General Electric, Boston, MA, USA). The resulting STL file was processed to create a 3D printable model while preserving anatomical detail. The model featured a root canal curvature of 40°, with an apical diameter of 0.20 mm and a 4% taper, corresponding to an ISO 20/.04 file. These parameters were selected to standardize the canal geometry for experimental purposes and do not represent the initial file size typically used in mesial canals of mandibular molars.

The model featured a root canal curvature of 40° and an initial apical diameter of 20.04 mm. It was printed using a high-precision resin-based 3D printer (Anycubic Technology Co., Shenzhen, China) to ensure geometric accuracy and repeatability. Only the mesial root of the printed tooth was used for the experimental procedures, and all printed replicas were identical to eliminate anatomical variability.

### 2.3. Canal Preparation and Fracture Evaluation

Three rotary nickel–titanium (NiTi) systems were evaluated: BlueShaper (Blue), BlueShaper Pro, and BlueShaper Gold (Zarc4Endo, Gijón, Asturias, Spain).

All three BlueShaper systems were manufactured from nickel–titanium (NiTi) alloy using distinct thermomechanical heat treatments. While the manufacturer does not disclose the precise parameters of these treatments, instruments referred to as “Blue-treated” are typically subjected to proprietary heat processes that raise the austenite finish (Af) temperature to between 35 and 45 °C, resulting in a martensitic or R-phase structure at body temperature. This phase composition is associated with increased flexibility and reduced stiffness. Conversely, “Gold-treated” instruments have Af values above 47 °C, corresponding to a more stable martensitic phase that improves fatigue resistance and ductility [25,26]. The BlueShaper Gold instrument was treated entirely using this “Gold” protocol. The BlueShaper (Blue) system underwent a standard Blue-wire heat treatment, while the BlueShaper Pro incorporated a dual-region configuration: the apical 4 mm exhibited Gold-phase behavior, whereas the remainder of the file retained characteristics consistent with Blue-treated martensitic NiTi. This dual configuration was verified using region-based material assignment in the FEA simulations.

All instruments were reported to share identical design characteristics, including a 16 mm active length, an apical diameter of 0.25 mm (Z4 file), and taper of 6%, a constant helical angle of 24°, and a convex triangular cross-section. To confirm the geometric consistency across systems and verify the presence of dual-phase treatment in BlueShaper Pro, three instruments from each group were examined using scanning electron microscopy (SEM) at 150× magnification at multiple axial levels (apical, middle, coronal). The evaluation focused on cross-sectional shape, flute configuration, helical design, and ensuring there were no visible surface discontinuities or changes in machining patterns between the apical (Gold-treated) and coronal (Blue-treated) portions of BlueShaper Pro.

Additionally, a representative instrument of each type was scanned using high-resolution micro-computed tomography (micro-CT), and the segmented models were exported as STL files. These STL datasets served as the base geometry for the 3D reconstruction of the instrument models used in the FEA. This approach ensured accurate preservation of the instruments’ dimensional and geometric characteristics without relying exclusively on manufacturer specifications.

All canal preparation procedures were conducted under simulated clinical conditions using continuous irrigation with 2.5% sodium hypochlorite (NaOCl). A total of 2 mL of NaOCl was introduced into the canal before each instrumentation cycle. Instrumentation was performed using a torque-controlled endodontic motor (Z-Evo, Zarc4Endo, Gijón, Asturias, Spain) operating at 500 rpm and 4 N·cm torque, following the manufacturer’s recommendations.

The total time to fracture (TTF) was recorded in seconds using a digital stopwatch. The number of rotations until fracture was calculated and used to determine the number of cycles to failure (NCF) calculated as NCF = (rpm × TTF/60). The number of canals successfully prepared by each instrument before fracture was also documented. Precisely, each instrument was used to prepare one canal at a time, in a continuous motion, until it reached full working length. If no fracture occurred, the same instrument was then used to prepare the next canal, and so on, until fracture was observed. However, the number of cycles per individual canal was not recorded, as the time taken to prepare each canal was not measured separately. Instead, the number of successfully prepared canals before fracture was used as a standardized performance metric, and the cumulative time until fracture was recorded continuously using a stopwatch. Following fracture, the length of the separated fragment was measured with a digital caliper (precision ±0.01 mm) (Mitutoyo Corporation, Aurora, IL, USA), and the location of the fracture was classified as occurring in the coronal (1), middle (2), or apical (3) third of the instrument. All measurements and classifications were confirmed under 4.5× magnification to ensure consistency and accuracy.

### 2.4. Finite Element Simulation

Three-dimensional models of BlueShaper, BlueShaper Pro, and BlueShaper Gold were constructed using Fusion 360^®^ (Autodesk Inc., San Francisco, CA USA). The base geometry of each instrument was obtained from high-resolution micro-CT scans of representative samples, which were segmented and exported as STL files to preserve external morphology and helical design. These STL models were imported into the CAD environment and digitally refined by cross-referencing with manufacturer-provided technical specifications, including apical diameter (0.25 mm), active length (16 mm), helical angle (24°), and cross-sectional shape. The integration process involved matching dimensional values to the CAD profile and verifying helical pitch and cross-section using both the micro-CT render and macro images under stereomicroscopy. Cutting and non-cutting zones were defined based on these validated reference points to ensure that the final model accurately represented the clinical instrument.

Material properties were defined according to the specific thermal treatment of each system. The BlueShaper model was assigned properties consistent with heat-treated martensitic (Blue Wire) NiTi: a Young’s modulus of 24 GPa, σ_start of 350 MPa, and σ_end of 550 MPa. The Gold model reflected stabilized martensite, with a Young’s modulus of 28 GPa, σ_start of 450 MPa, and σ_end of 650 MPa. For the BlueShaper Pro (Dual Wire), the apical 4 mm was modeled using NiTi with gold heat-treated properties, while the rest of the instrument used the characteristics of the blue treatment, implemented through region-based material assignment in ANSYS.

This dual-material assignment allowed a realistic simulation of the hybrid behavior of the Pro system under torsional, flexural, and fatigue conditions. All other simulation parameters, including boundary conditions and meshing strategies, were kept identical across systems for comparative consistency.

The simulations were conducted using the Static Structural module of ANSYS Workbench 2023 R1 (Ansys Inc., Canonsburg, PA, USA). Each NiTi instrument was assigned specific mechanical properties based on its alloy treatment. For BlueShaper (Blue Wire), a Young’s modulus of 24 GPa, Poisson’s ratio of 0.30, and density of 6.45 g/cm^3^ were used, reflecting its stable martensitic behavior. BlueShaper Gold (Gold Wire) was modeled with a higher modulus of 28 GPa and similar Poisson’s ratio and density, representing its austenitic composition stabilized by heat treatment. For BlueShaper Pro (Dual Wire), a region-based material assignment was implemented: the apical 4 mm used Gold-treated NiTi properties, and the remaining instrument length used Blue-treated NiTi values. This dual-region configuration aimed to replicate the hybrid metallurgical structure of the instrument.

Fatigue behavior was modeled using a strain–life (ε–N) approach, with values specific to each alloy’s treatment [16]. Although all simulations were run under linear elastic assumptions to ensure computational stability, the material property variations were incorporated to reflect the distinct biomechanical behavior of each system under torsional, flexural, and cyclic loads. This approach allowed for comparative assessment of stress distribution, deformation patterns, and fatigue-related variables while preserving the influence of alloy treatment on each instrument’s performance.

The dentin model, corresponding to the mesial root of a mandibular molar with 40° curvature, was meshed separately with an element size of 0.2 mm using a quadrilateral-dominant sweep method. Dentin properties were defined as isotropic: a Young’s modulus 18.6 GPa, Poisson’s ratio 0.31, and density 2.0 g/cm^3^. Canal geometry was derived from the same 3D-printed root canal model used for experimental testing, ensuring alignment between in vitro and simulated conditions.

**Figure 1:**
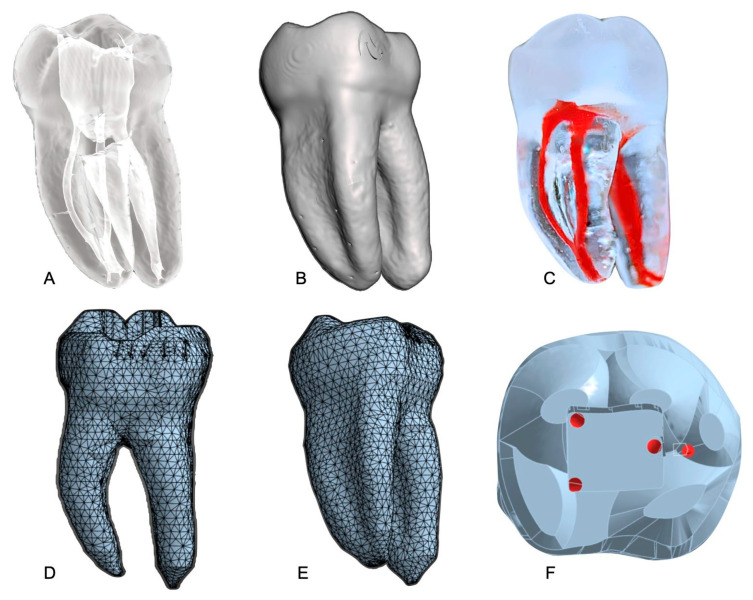
Workflow for anatomical modeling and simulation. (**A**) STL model of the 3D-printed tooth replica generated from micro-CT imaging. (**B**) External view of the same STL model illustrating the root curvature and dentin morphology. (**C**) Photograph of the final 3D-printed replica showing the internal canal configuration filled with red hydrogel for visual contrast. (**D**) Finite element mesh of the tooth model (buccal view) used to simulate instrument–dentin interactions. (**E**) Alternate view of the meshed model (lingual view) highlighting the apical region. (**F**) Schematic representation of the applied moment and loading points within the mesial canals during finite element analysis.

Four distinct FEA simulations were conducted: cyclic fatigue, torsional, flexural, and instrument–tooth interaction simulations. The workflow for anatomical modeling is shown in Figure 1.

### 2.5. Cyclic Fatigue Simulation

A fatigue tool was implemented under zero-based loading, using von Mises equivalent stress as the critical variable. Fatigue life, damage, biaxiality indication, safety factors, and alternating stress distribution were calculated without correction for mean stress. This enabled the estimation of the NCF under clinically relevant rotation.

### 2.6. Torsional Simulation

Torsional loading was applied by fixing the apical 3 mm of the instrument and exerting a 40 N·mm moment on the shaft, thereby reproducing the manufacturer’s recommended clinical torque. The model provided von Mises stress, shear stress, total deformation, and directional deformation, which were used to assess the stress concentration in the instrument’s core.

### 2.7. Flexural Simulation

To simulate oblique flexion, a ramped force was applied with components of −2 N (X), 5 N (Y), and −10 N (Z), mimicking a bending angle of 17.5°. The apical 3 mm was fixed, and the results included total and directional deformation, shear and von Mises stress, and equivalent elastic strain, highlighting regions prone to structural deformation.

### 2.8. Instrument–Tooth Interaction Simulation

The same 3D molar model used in clinical experiments was employed to evaluate instrument–dentin contact during simulated shaping. The model involved instrument insertion within the canal and provided output data on contact pressure, stress distribution, and deformation within the dentin walls. Mesh convergence tests were conducted in all simulations to ensure accuracy, and boundary conditions were consistent across all instruments to facilitate comparative analysis.

The data generated from the FEA simulations were exported for quantitative comparison across all systems. Key parameters—including von Mises stress, shear stress, total deformation, directional deformation, and equivalent elastic strain—were extracted from each model under identical loading conditions. The predicted NCF, damage distribution, and safety factors for the fatigue module were recorded for each material region.

To ensure consistency, all measurements were taken at the same apical, middle, and coronal locations across instruments and tooth models, enabling a spatial correlation of stress concentration with known fracture-prone zones. The values obtained from FEA were then compared with those from mechanical testing and clinical replica instrumentation. Additionally, contact pressure and stress intensity maps were visually inspected to identify differences in instrument–dentin interaction. This comprehensive analysis facilitated data triangulation from physical, virtual, and clinical models, thereby strengthening the biomechanical interpretation of each system’s performance.

### 2.9. Mechanical Testing

Mechanical testing was performed using a universal testing machine (INSTRON 3345, Instron, Norwood, MA, USA) equipped with a custom-fabricated stainless-steel device designed to simulate standardized canal curvature. The device featured artificial canals with a width of 1.5 mm, a total length of 26 mm, and a curvature of 40° with a 3 mm radius, intended to impose consistent mechanical stress on rotary NiTi instruments during testing. This setup enabled the assessment of mechanical performance parameters, including minimum and maximum load, TTF, and NCF. The testing machine offered a precision of ±0.5%, a force measurement range from 0.1 N to 5 kN, and a maximum crosshead speed of 1000 mm/min.

All procedures were performed under wet conditions using 2.5% NaOCl as the irrigant, with 2 mL introduced into the canal before each preparation to maintain lubrication and simulate clinical conditions. A transparent acrylic lid was placed over the canal block to enable direct visual monitoring of file progression and fracture events.

Each file was mounted in a 6:1 reduction contra-angle handpiece (WaveOne, Dentsply-Maillefer, Ballaigues, Switzerland) and operated by a cordless endodontic motor fixed to a vertical support attached to the universal testing machine. The handpiece was aligned to maintain a standardized trajectory relative to the curvature of the artificial canal. Instrumentation was performed in a single, uninterrupted insertion until spontaneous fracture occurred.

To prevent direct contact with the metallic canal entrance, the file was initially positioned 0.5 mm above the canal orifice. To better simulate clinical conditions and ensure continuous interaction with intracanal tissue, the instrument was introduced while already rotating, rather than being placed statically at full working length. Despite the coronal canal diameter (1.5 mm) being larger than the file diameter at D16 (1.2 mm), this approach enabled a controlled and progressive engagement. File advancement was driven by the crosshead of the testing machine, moving linearly at a constant speed of 23.5 mm/min, as established in previous studies to ensure experimental stability. This rate was selected based on the prior literature and experimental stability [14], providing consistent engagement with the canal curvature without introducing excessive mechanical artifacts. The file rotated continuously at 500 rpm with a torque limit of 4 N·cm, in accordance with the manufacturer’s specifications. Time to fracture (TTF) was recorded using a stopwatch, and the number of cycles to failure (NCF) was calculated as NCF = (rpm × TTF/60).

### 2.10. Statistical Analysis

Statistical analysis was conducted using SPSS (version 28.0, IBM Corp., Armonk, NY, USA). Descriptive statistics (mean ± SD) were calculated for all variables. Normality was evaluated with the Shapiro–Wilk test, and homogeneity of variances was checked using Levene’s test. Since the results indicated a normal distribution, one-way ANOVA followed by Tukey’s post hoc test was performed. A *p*-value < 0.05 was considered statistically significant.

Statistical tests were not applied to the data obtained from finite element analysis, as these simulations are deterministic and not subject to random variation. Each FEA result represents a controlled, repeatable output based on defined boundary conditions, material properties, and geometric parameters. Therefore, comparisons between systems were made descriptively using the numerical values of key mechanical indicators (e.g., von Mises stress, shear stress, deformation) and visual inspection of stress distribution maps. These outcomes were interpreted in the context of the physical and clinical findings to support biomechanical conclusions.

## 3. Results

A total of 60 instruments were evaluated—20 per group (BlueShaper, BlueShaper Pro, and BlueShaper Gold)—across three experimental settings: simulated clinical use with 3D-printed replicas, mechanical resistance testing, and FEA analyses.

### 3.1. Clinical Performance in 3D-Printed Replicas

The results of the clinical performance are presented in Table 1. The number of canals prepared before fracture showed significant differences between groups (*p* < 0.001), with BlueShaper Gold demonstrating the highest endurance (7.47 ± 0.99), followed by BlueShaper Pro (5.67 ± 1.05) and BlueShaper (5.00 ± 0.76).

TTF also differed significantly (*p* < 0.05), with Gold showing longer survival (39.0 ± 6.7 s) compared to Pro (32.2 ± 6.8 s) and Blue (30.5 ± 5.9 s). The calculated NCF reflected the same trend (Gold: 325 ± 55.7; Pro: 268.6 ± 56.9; Blue: 258 ± 55; *p* < 0.05).

There were no significant differences in fracture location (primarily the apical third for all groups; *p* > 0.05) or fragment length (mean ≈ 5 mm, *p* > 0.05).

### 3.2. Mechanical Testing

The findings from mechanical testing are presented in Table 1. The overall mean minimum load was 3.42 ± 1.81 N. BlueShaper Gold exhibited the highest initial resistance (4.22 ± 2.35 N), followed by Blue (3.57 ± 1.39 N) and Pro (2.74 ± 1.52 N). Statistical analysis revealed significant differences (*p* < 0.001), with Pro performing significantly worse than both Gold (*p* < 0.001) and Blue (*p* < 0.05).

The average maximum load was 5.12 ± 2.54 N. Gold instruments withstood significantly higher loads (6.08 ± 3.08 N) than Pro (4.34 ± 2.76 N; *p* < 0.05). The Blue group (5.06 ± 2.12 N) did not differ significantly from the other groups.

Pro instruments exhibited the longest TTF (73.85 ± 7.10 s), followed by Gold (67.45 ± 8.35 s) and Blue (65.62 ± 5.08 s). Differences were statistically significant (*p* < 0.001), with Pro showing superior fatigue resistance compared to both other groups (*p* < 0.001).

### 3.3. Finite Element Analysis

**Figure 2:**
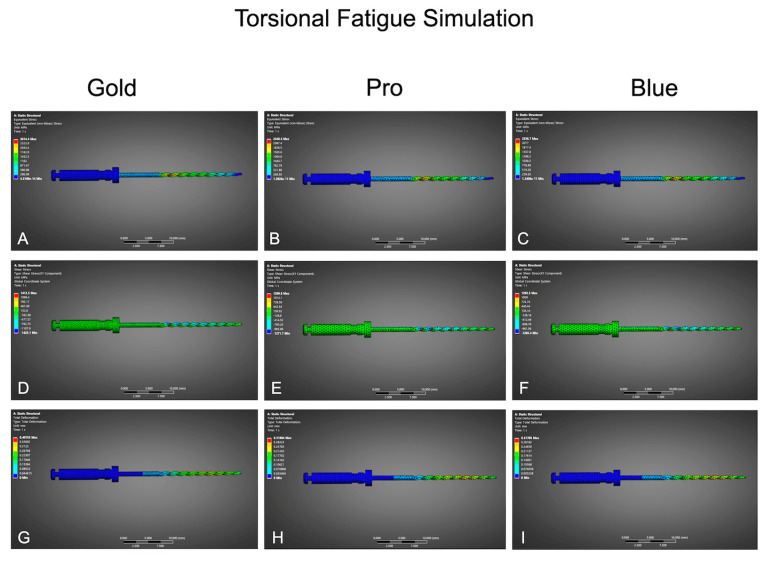
Finite element analysis of torsional fatigue simulation for BlueShaper Gold (left column), BlueShaper Pro (center column), and BlueShaper Blue (right column). (**A**–**C**) Equivalent von Mises stress distributions (MPa), illustrating areas of maximum stress concentration during torsional loading. (**D**–**F**) Shear stress maps (MPa) reflecting the magnitude of internal forces contributing to torsional deformation. (**G**–**I**) Total deformation results (mm) highlighting displacement patterns under torsional stress.

Under simulated torsion (Table 2, Figure 2), BlueShaper Pro exhibited the highest von Mises stress (2890 MPa) and shear stress (1575.5 MPa), followed by Blue (2735.0 MPa, 1521.3 MPa) and Gold (2614.4 MPa, 1413.5 MPa). Total deformation values were moderate for Gold (0.3055 mm), slightly higher for Pro (0.314 mm), and slightly lower for Blue (0.298 mm), suggesting that Gold optimally balances flexibility with structural integrity.

**Figure 3:**
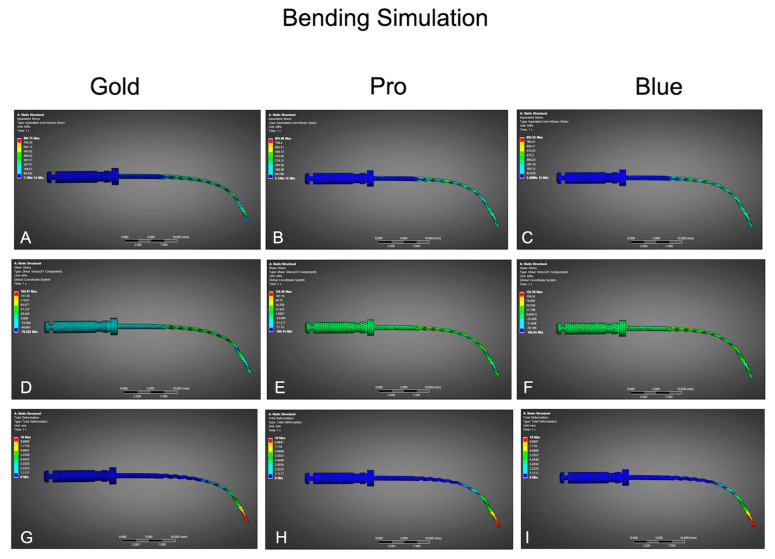
Finite element analysis results of bending simulation for the three tested instruments: BlueShaper Gold (left column), BlueShaper Pro (center column), and BlueShaper Blue (right column). (**A**–**C**) Equivalent von Mises stress distribution under simulated bending load. (**D**–**F**) Principal stress components (Type 1) illustrating tensile stress zones along the instrument shaft. (**G**–**I**) Total deformation maps indicating displacement under bending conditions.

Simulations under bending conditions (Table 3, Figure 3) revealed notable differences in mechanical response among the systems. BlueShaper Gold exhibited the lowest peak von Mises stress (5771.1 MPa) and equivalent elastic strain (0.02887 mm/mm), followed closely by BlueShaper Pro (5869.0 MPa, 0.02936 mm/mm). BlueShaper presented the highest values (5935.3 MPa, 0.03012 mm/mm), indicating a poorer behavior under flexural stress. Total deformation was also highest for Gold (11.566 mm), slightly lower for Pro (11.414 mm), and lowest for Blue (10.985 mm).

**Figure 4:**
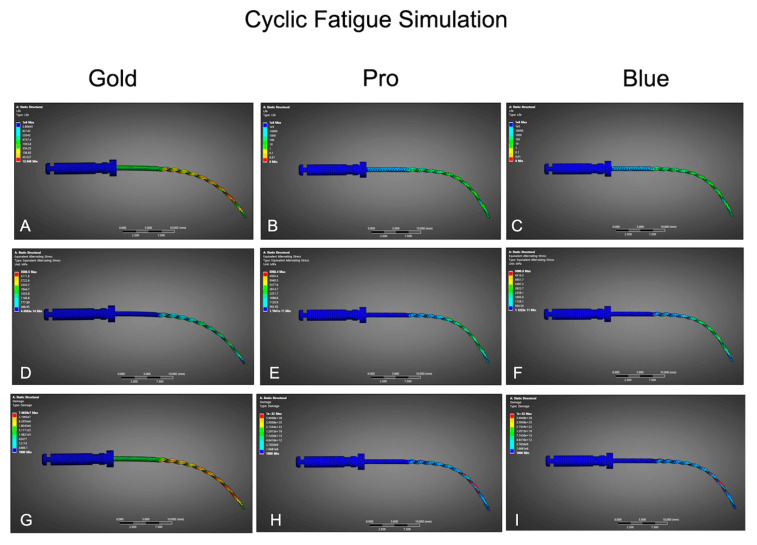
FEA results of cyclic fatigue simulation for BlueShaper Gold (left column), BlueShaper Pro (center column), and BlueShaper Blue (right column). (**A**–**C**) Predicted lifespan (cycles to failure) under simulated cyclic loading conditions. (**D**–**F**) Equivalent alternating stress distribution maps (MPa), illustrating areas of highest fatigue-related stress. (**G**–**I**) Damage factor maps, where values approaching 1 indicate higher risk of failure due to accumulated fatigue.

Under simulated cyclic loading conditions (Table 2, Figure 4), BlueShaper Gold demonstrated the most favorable fatigue profile, featuring the lowest alternating equivalent stress (623.5 MPa), the lowest damage factor (0.21), and the highest predicted NCF (1337 cycles). BlueShaper Pro exhibited a higher fatigue stress (684.2 MPa), a damage factor of 0.32, and an estimated lifespan of 1175 cycles. BlueShaper displayed the highest cyclic stress (701.3 MPa), the greatest damage accumulation (0.38), and the shortest predicted fatigue life (1083 cycles). These numerical results align closely with the actual NCF recorded in the clinical replica tests, which supports the predictive validity of the fatigue simulation model.

**Figure 5:**
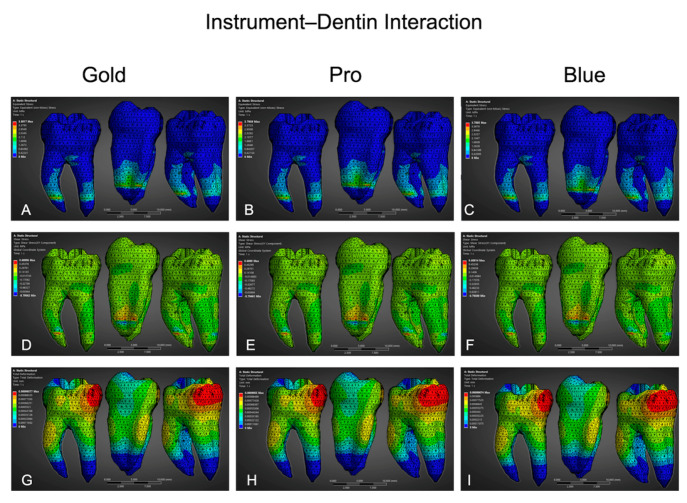
FEA results of instrument–dentin interaction for BlueShaper Gold (left column), BlueShaper Pro (center column), and BlueShaper Blue (right column) during simulated canal shaping. (**A**–**C**) Equivalent von Mises stress (MPa) in dentin, illustrating areas of peak compressive and tensile stress. (**D**–**F**) Shear stress (MPa) distribution, representing tangential forces exerted by the instruments on canal walls. (**G**–**I**) Total deformation (mm), indicating localized dentin displacement under simulated mechanical loading.

The instrument–dentin interaction (Table 4, Figure 5) analyses revealed measurable differences among the systems. BlueShaper (Blue) demonstrated the highest von Mises stress (3.41 MPa), followed closely by Pro (3.40 MPa) and Gold (3.42 MPa). Despite the close values, Pro exhibited slightly more localized stress concentration along the inner curvature of the apical third. Regarding shear stress, all systems showed nearly identical peak values: Blue (0.498 MPa), Pro (0.498 MPa), and Gold (0.497 MPa), indicating comparable lateral cutting pressure during simulated instrumentation.

Total deformation values were identical across all instruments (0.00091 mm), indicating minimal but measurable dentin displacement under simulated mechanical interaction.

## 4. Discussion

This study employed a comprehensive multimodal approach to evaluate the performance of three rotary NiTi systems—BlueShaper (Blue), BlueShaper Pro (Dual Wire), and BlueShaper Gold—by combining mechanical testing, FEA, and simulated clinical instrumentation using 3D-printed mandibular molar replicas. Despite identical geometries, the three systems exhibited distinct biomechanical responses, highlighting the influence of metallurgical treatment on fatigue resistance, stress distribution, and clinical performance.

In the simulated clinical model, BlueShaper Gold prepared significantly more canals before fracture (7.5 ± 0.99), outperforming BlueShaper Pro by 32% and Blue by 50%. The number of cycles followed the same trend: Gold (325 ± 55.7), Pro (268.6 ± 56.9), and Blue (258 ± 55).

The behavior of NiTi instruments under clinical and mechanical stress is closely related to their phase transformation temperatures. Although specific DSC data for BlueShaper instruments are not available, prior studies have shown that Blue-treated NiTi typically presents an Af between 35 and 45 °C, resulting in a predominantly martensitic or R-phase structure at body temperature [7,26]. In contrast, Gold-treated alloys often exhibit Af values above 47 °C, leading to a more stable martensitic phase under clinical conditions, which enhances ductility and fatigue resistance [25,26]. BlueShaper Pro combines both treatments in a dual configuration, with a Gold-treated apical region and a Blue-treated shaft. This configuration likely contributes to its intermediate behavior, balancing flexibility and structural stability. These differences in thermal behavior explain the improved performance observed in the Gold and Pro instruments in our study.

These findings align with previous studies showing improved fatigue resistance in thermally treated Gold alloys due to enhanced martensitic phase stability and ductility [6,26,27,28,29].

It is important to clarify that the terms “Blue” and “Gold” do not indicate differences in alloy composition. Instead, they refer to the surface oxide layer coloration that results from distinct proprietary thermal treatments applied to the same NiTi base alloy. These heat treatments modify the phase transformation behavior and mechanical properties of the file, and the resulting color, blue or gold, is a visual byproduct of the specific oxide layer formed during the process.

Although several regulatory guidelines discourage or prohibit the clinical reuse of rotary NiTi instruments, the number of canals prepared until fracture in this study was not intended to support or suggest multiple use in clinical practice. Rather, this metric was employed as a standardized method to compare the endurance and fatigue resistance of instruments under controlled conditions. Evaluating how many canals an instrument can prepare before failure, while maintaining identical anatomical and operational parameters, offers valuable insight into its structural resilience and failure threshold. These findings help differentiate alloy behavior and fatigue performance, particularly in systems that share identical geometry but differ in thermal treatment. Therefore, the data presented here should be interpreted as a measure of relative durability, not as a clinical recommendation for reuse.

BlueShaper Pro, although not the strongest in terms of maximum fracture resistance, exhibited the longest TTF (73.9 ± 7.1 s), suggesting that its dual-wire configuration, combining the flexibility of Blue-treated NiTi alloy along the shaft and the cutting efficiency of a Gold-treated tip, helps dissipate cyclic stresses more effectively. The Gold-treated apical portion likely presents a more stable martensitic phase at body temperature, which favors increased flexibility and resistance to crack initiation. In contrast, the Blue-treated shaft maintains a mixed austenite–martensite composition, which contributes to greater stiffness and torsional stability. This metallurgical design may serve as a mechanical buffer, delaying microcrack propagation during repetitive motion, which can be advantageous in retreatments or extended shaping procedures.

Mechanical testing reinforced these results. BlueShaper Gold recorded the highest minimum (4.22 ± 2.35 N) and maximum load (6.08 ± 3.08 N), surpassing Pro by 54% and Blue by 40%, respectively. BlueShaper (Blue) displayed moderate static fracture resistance (5.06 ± 2.12 N), but showed a shorter time to failure under cyclic loading, limiting its suitability in highly curved canals where fatigue resistance is critical. In such cases, instruments with greater endurance under repeated stress, rather than just higher fracture thresholds, may be preferable for safe single use [30,31]. These values are consistent with the mechanical implications of the respective thermal treatments: the Gold-treated NiTi instrument, with its higher martensitic content, provides enhanced ductility and resistance to torsional overload, whereas the Blue-treated NiTi instrument, although more flexible, shows earlier structural compromise under load.

The present findings align with prior studies on size 25 files with 6% taper [32,33,34]. Topçuoğlu et al. [33] evaluated ProTaper Next X2 (25/.06, Dentsply Sirona, Ballaigues, Switzerland), HyFlex CM (25/.06, Coltène/Whaledent, Altstätten, Switzerland), and OneShape (25.06, Micro-Mega, Besançon, France) in a double-curved canal and reported significantly higher fatigue resistance for heat-treated files, such as ProTaper Next X2 and HyFlex CM, compared to conventional NiTi designs. This aligns with the present observation that thermally treated files, like Gold and Pro, perform better than standard Blue.

Similarly, a previous study compared ProTaper Next X2, 2Shape (25/.06, Micro-Mega, Besançon, France), HyFlex CM, and TF Adaptive (25/.06, Kerr Endodontics, Orange, CA, USA), all with 25/.06 geometry, and found a consistent fatigue resistance hierarchy linked to alloy conditioning, with HyFlex CM and TF Adaptive outperforming ProTaper Next X2 and 2Shape [32]. These trends are in line with the present results, where differences in thermal treatment, rather than geometry, primarily influenced endurance. Overall, these comparisons confirm that metallurgical conditioning plays a dominant role in fatigue behavior, and that the performance of BlueShaper Gold and Pro aligns with patterns observed in other commercially available thermally treated systems.

The strong agreement between FEA simulations and both mechanical and clinical results reinforces the predictive power of computational models in endodontics. For instance, Gold’s lowest von Mises stress (2614 MPa), lowest shear stress (1413.5 MPa), and greatest torsional deformation (0.305 mm) correlated directly with its superior resistance in both cyclic fatigue testing (1337 predicted cycles, 325 NCF) and real canal instrumentation (7.5 canals prepared). These results are consistent with its gold heat treatment, which stabilizes the martensitic phase and enhances ductility under torsional and cyclic loading. A material predominantly in the martensitic state at body temperature tends to exhibit greater capacity to deform without fracturing, as reflected in the FEA output. Pro, with the highest von Mises (2890 MPa) and shear stress (1575.5 MPa), demonstrated the longest fracture time (73.9 s), suggesting a stress distribution pattern that delays failure. This may reflect the influence of its dual-phase structure, which modifies stress propagation patterns, delaying the onset of catastrophic failure. Blue’s intermediate values in stress and deformation are aligned with its lower fatigue life and performance. The limited phase transformation capacity under stress may contribute to earlier accumulation of damage. These convergent results support the use of FEA as a reliable tool for forecasting clinical behavior, particularly when paired with physical testing and simulated models.

An additional observation from the simulated clinical tests was that the length of the fractured segment was consistently similar across all three instrument groups, with no statistically significant differences. This suggests that the presence of a dual thermal treatment in the BlueShaper Pro, featuring a Gold-treated tip and a Blue-treated shaft, did not predispose the instrument to earlier fracture at the transitional zone. If the interface between the two thermally treated regions of the same NiTi alloy had acted as a mechanical weak point, a shorter fragment length (i.e., closer to the tip) or preferential fracture at the transition zone would have been expected; however, this was not observed. This interpretation is further supported by the finite element simulations, which did not show increased stress accumulation in the apical region of BlueShaper Pro under torsional, flexural, or cyclic loading conditions. These findings suggest that the transition between Gold and Blue-treated NiTi instruments in the Dual Wire configuration does not compromise structural integrity or concentrate stress in clinically relevant scenarios.

The cyclic fatigue simulation closely mirrored the experimental trends, reinforcing the reliability of FEA as a preclinical evaluation tool [17,18]. BlueShaper Gold demonstrated the highest predicted fatigue life (1337 cycles), outperforming Pro and Blue by 13.7% and 23.4%, respectively. Correspondingly, Gold exhibited the lowest damage factor (0.21), followed by Pro (0.32) and Blue (0.38), suggesting reduced microstructural fatigue accumulation. These findings align with real-world outcomes in both mechanical testing and 3D-printed canal simulations, supporting the accuracy of digital models in forecasting clinical performance [18,22,24]. Clinically, instruments with lower damage accumulation are less likely to fracture prematurely, particularly in curved and challenging anatomies [7,35]. This indicates that Gold may offer greater procedural safety, while Pro’s hybrid profile supports extended use in anatomically complex cases. The strong correlation between FEA results and empirical data validates the integration of simulation in modern endodontic research and highlights its value in guiding both instrument selection and future design optimization.

The finite element simulations evaluating the action of the instrumentation systems on the tooth revealed comparable patterns of stress and deformation across all three groups. While minor variations in von Mises stress, shear stress, and total deformation were observed, the overall distribution of mechanical load in the dentin was similar among Gold, Pro, and Blue instruments. This suggests that the three instruments generate comparable mechanical responses in the dentin structure when subjected to identical load conditions in a standardized canal anatomy.

Although BlueShaper Pro demonstrated a slightly more balanced stress distribution in the apical third, and Blue exhibited marginally higher total deformation, these differences were not substantial enough to definitively favor one system over the others based solely on the FEA tooth interaction analysis. Instead, the simulation results provide a visual and quantitative complement to the mechanical testing and 3D replica performance outcomes, which more clearly highlighted the variations in cyclic fatigue resistance, fracture behavior, and shaping ability.

Therefore, the FEA analysis supports the clinical and experimental findings by confirming that material properties and internal alloy configurations do not lead to drastic changes in external dentin stress patterns under controlled conditions [36,37]. The similarity in stress maps reinforces the idea that all three systems are mechanically safe for use in curved canals, and that distinctions in performance likely arise more from internal instrument behavior (e.g., stress accumulation and dissipation) than from their direct impact on surrounding dentin.

From a cost-effectiveness perspective, BlueShaper Gold may improve safety in complex procedures thanks to its superior fatigue resistance, but its advanced thermal treatment makes it the most expensive option. BlueShaper Pro, with its hybrid design and intermediate durability, provides a balanced compromise between performance and cost. In contrast, the standard BlueShaper is more affordable but demonstrated lower resistance, which may lead to more frequent replacements. Therefore, selecting the appropriate instrument requires weighing mechanical performance against cost-efficiency in light of specific clinical demands.

Although 4% taper instruments are often recommended for curved mesial canals to minimize dentinal stress [8,38], several clinical and experimental studies have shown that 25/.06 files can be safely and effectively used in these anatomies when a glide path is established and shaping protocols are respected [39,40,41]. Moreover, the 25/.06 configuration is widely adopted in mechanical and FEA studies for its ability to generate reproducible stress patterns and highlight performance differences between file systems [39,40,41]. For these reasons, it remains a valid and justified choice for evaluating the mechanical behavior of NiTi instruments under standardized conditions.

Despite the strengths of this study, certain limitations exist. While 3D-printed replicas were generated from high-resolution micro-CT scans and standardized across groups, they cannot fully replicate the mechanical heterogeneity of natural dentin, such as hydration, microcracks, or viscoelastic behavior. Additionally, the absence of irrigation dynamics, thermal effects, and operator variability limits the direct translation of these results to clinical scenarios. While FEA offers useful information on stress distribution and deformation, it remains a simplified model. Assumptions of homogeneous materials and ideal boundary conditions do not fully reflect the complex behavior of NiTi in vivo. FEA also excludes clinical variables like manufacturing defects, canal anatomy, and irrigation. Thus, its predictions should be interpreted in conjunction with experimental validation.

Future studies should integrate real-time cutting analysis, thermal fatigue behavior, and canal shaping patterns in extracted teeth to further validate these findings.

In conclusion, this study demonstrates that BlueShaper Gold provides superior mechanical strength and fatigue resistance under standardized conditions, suggesting it may offer advantages in procedures involving moderate to severe curvatures. BlueShaper Pro exhibited a well-balanced performance, combining apical cutting efficiency with coronal flexibility, which may be beneficial in extended instrumentation protocols. Although structurally consistent, BlueShaper (Blue) exhibited lower fatigue resistance and higher stress concentration, suggesting that its use may be more suitable for final apical enlargement or cases with reduced curvature demands. While only a single canal anatomy was tested, the results highlight the importance of case-specific instrument selection and support the integration of mechanical, clinical, and computational evaluations in endodontic research.

## 5. Conclusions

Despite identical design, NiTi instruments with different thermal treatments showed significant variations in fatigue resistance and stress behavior. These findings support the use of alloy-based instruments to enhance safety and performance in anatomically challenging root canal procedures.

## Figures and Tables

**Table 1:** Clinical and mechanical performance of three BlueShaper systems: Blue, Pro (Dual Wire), and Gold expressed as mean ± standard deviation.

Parameter	BlueShaper (Blue)	BlueShaper Pro (Dual Wire)	BlueShaper Gold
Canals Prepared (mean ± SD)	5.00 ± 0.76	5.67 ± 1.05	7.5 ± 0.99
NCF	258 ± 55	268.6 ± 56.9	325 ± 55.7
TTF (s)	65.6 ± 5.1	73.9 ± 7.1	67.4 ± 8.3
Fracture Location (Apical, %)	93%	93%	93%
Fragment Length (mm)	4.92 ± 0.54	4.87 ± 0.74	5.00 ± 0.53
Minimum Load (N)	3.57 ± 1.39	2.74 ± 1.52	4.22 ± 2.35
Maximum Load (N)	5.06 ± 2.12	4.34 ± 2.76	6.08 ± 3.08
Fracture Time in Instron (s)	65.62 ± 5.08	73.85 ± 7.10	67.45 ± 8.35

Abbreviations: SD, standard deviation; NCF, number of cycles to fracture; TTF, time to fracture.

**Table 2:** FEA results showing the torsional stress response and simulated fatigue life and damage accumulation of three NiTi systems.

Group	Von Mises Stress (MPa)	Shear Stress (MPa)	Total Deformation (mm)	Fatigue Life (Cycles)	Damage Factor
BlueShaper	2735	1521.7	0.2541	1083	0.38
BlueShaper Pro	2890	1575.5	0.2893	1175	0.32
BlueShaper Gold	2614.4	1413.5	0.3055	1337	0.21

**Table 3:** FEA results from bending simulation of three NiTi rotary instruments, showing internal stress and deformation parameters under flexural loading.

Group	Von Mises Stress (MPa)	Stress Intensity (MPa)	Shear Stress (MPa)	Equivalent Elastic Strain (mm/mm)	Directional Deformation (mm)	Total Deformation (mm)
BlueShaper	5771.1	5833.8	1631.8	0.0289	0.2052	11.414
BlueShaper Pro	5869.0	5930.0	1653.0	0.0294	0.2093	11.566
BlueShaper Gold	5593.0	5660.0	1548.0	0.0272	0.2022	11.878

**Table 4:** FEA results of dentin–instrument interaction during simulated shaping, showing contact stress and deformation patterns for the three NiTi systems.

Group	Von Mises Stress (MPa)	Shear Stress (MPa)	Total Deformation (mm)
BlueShaper	3.41	498	0.01
BlueShaper Pro	3.4	498	0.01
BlueShaper Gold	3.42	497	0.01

## Data Availability

The data supporting this study’s findings are available from the corresponding author upon reasonable request.
